# Death of Dr. E. H. Elliott

**Published:** 1871-02

**Authors:** 


### Death of Dr. E. H. Elliott.

At the last semi-annual meeting of the Niagara County Medical Society, the President appointed Drs. Gould and Faling a committee to draw up resolutions on the death of Dr. Edward H. Elliott, of Hartland, N. Y.

The report of the committee, which was accepted, is as follows:

Whereas, It has pleased Almighty God, in his all-wise providence, to remove by death, one of our much loved members, in the person of Dr. E. H. Elliott,

Resolved, That the society learns with unfeigned sorrow of the death of Dr. E. H. Elliott, one of its most worthy members.

Resolved, That in the death of said member the profession loses a brother in whom the many virtues of a good physician, true friend and worthy citizen were most conspicuous.

Resolved, That while we mourn his loss, we are reminded of our own mortality, and that it behooves us, as it did our worthy brother, in the midst of life, with its cares, perplexities and uncertainties to prepare for the solemn hour of our departure.

Resolved, That we tender to the family and relatives of our deceased brother our heartfelt sympathy in their bereavement, and ever pray that the blessing of the widow's God, and the father of the fatherless may rest upon them.

WM. B. GOULD,
PETER FALING.

